# Serum fatty acid binding protein 4 levels are associated with abdominal aortic calcification in peritoneal dialysis patients

**DOI:** 10.1080/0886022X.2021.2003205

**Published:** 2021-11-17

**Authors:** Sijia Zhou, Xiaoxiao Wang, Junbao Shi, Qingfeng Han, Lian He, Wen Tang, Aihua Zhang

**Affiliations:** a Department of Nephrology, Peking University Third Hospital, Beijing, China; b Research Center of Clinical Epidemiology, Peking University Third Hospital, Beijing, China; c Department of Nephrology, Xuanwu Hospital Capital Medical University, Beijing, China

**Keywords:** Adipokine, FABP4, peritoneal dialysis, vascular calcification, CKD-MBD

## Abstract

**Background:**

Fatty acid binding protein 4 (FABP4) is an adipokine that was mainly derived from adipocytes and macrophages. Vascular calcification (VC) is highly prevalent in peritoneal dialysis (PD) patients and could predict their cardiovascular mortality. The pathogenesis of VC is complex, and adipokines may play an important role in it. This study aimed to examine the relationship between serum FABP4 and VC in PD patients.

**Methods:**

Serum FABP4 was measured by enzyme-linked immunosorbent assay. According to the median value of serum FABP4, the participants were divided into the low FABP4 group and the high FABP4 group. Lateral plain X-ray films of abdomen were used to evaluate the abdominal aortic calcification (AAC) score. The participants were divided into the high AAC score group (AAC score ≥4, indicating moderate or heavy VC) and the low AAC score group (AAC score <4, indicating no or mild VC).

**Results:**

116 PD patients were involved in the study. The AAC score and the proportion of patients with an AAC score ≥4 of the high FABP4 group were significantly higher than those of the low FABP4 group. Serum FABP4 of the high AAC score group was significantly higher than that of the low AAC score group [164.5 (138.4, 362.8) ng/mL versus 144.7 (123.8, 170.1) ng/mL, *p* = 0.002]. Serum FABP4 was positively associated with the AAC score according to the multivariate linear regression analysis. In the multivariate logistic regression analysis, serum FABP4 was the independent influencer of an AAC score ≥4.

**Conclusions:**

Serum FABP4 is positively associated with the AAC score and is an independent marker of AAC in PD patients.

## Introduction

Vascular calcification (VC) is the key element of chronic kidney disease mineral bone disease (CKD-MBD). An increasing body of evidence suggests that VC is a predominant cause of cardiovascular diseases and could increase the cardiovascular mortality in patients with end-stage renal disease (ESRD) [1], especially in patients on maintenance dialysis. However, the overall pathogenesis of VC is still unclear, and the optimal interventions are limited. It is important to find out novel biomarkers and potential treating targets to improve the cardiovascular outcome of patients on dialysis.

An increase of body fat is common in peritoneal dialysis (PD) patients [2], which is associated with the proinflammatory state and the alteration in lipid profile [3]. Current researches suggest that fat tissue plays an important role in modulating lots of complications among PD patients, mainly through secreting various adipokines [4]. Fatty acid binding protein 4 (FABP4), also known as adipocyte FABP (A-FABP) or adipocyte protein 2 (aP2), is abundantly expressed in adipocytes and activated macrophages [5]. Adipokines may affect intimal arterial calcification in PD patients through regulating atherosclerosis. Studies also showed positive associations between serum FABP4 and other cardiovascular risk factors like obesity, dyslipidemia, and insulin resistance [6–8]. Medial arterial calcification is specific in patients with CKD-MBD, and the transformation of vascular smooth muscle cells into osteoblast-like cells is recognized as a key process in VC. There are many complex cross-talks between adipocytes, vessels, and bone tissues in patients on dialysis. Recent studies found that adipokines may influence the bone metabolism [9,10]. A basic study [11] reported that inhibition of FABP4 could alleviate osteoarthritis induced by high-fat diet in mice. These data corroborate the fact that FABP4 may play an important role in the development of VC among PD patients. However, no research has investigated the relationship between FABP4 and VC.

We hypothesize that levels of circulating FABP4 may influence the process of VC in PD patients. To test the hypothesis, we investigated the association of serum FABP4 and the presence of abdominal aortic calcification (AAC) in PD patients, then examined the main related factors of the AAC score. Our study would provide more evidence of the impacts of adipokines on VC in patients on dialysis.

## Materials and methods

### Patients

This cross-sectional study was conducted in Peking University Third Hospital between July and October 2016. 159 PD patients were screened based on the following inclusion and exclusion criteria: 18 years or older, stably treated with PD for at least 6 months, agreed to participate in the study, and no sign of clinically active infections or some other acute complications. Finally, 116 PD patients were recruited. All participants received four exchanges per day following a standard regimen (8 L/day).

All participants had provided their informed consent before participating in the study. The study protocol was approved by the Ethics Committee of the Peking University Third Hospital (M2020181) and performed according to the Declaration of Helsinki.

### Clinical parameters, biochemical assays, and assessment of serum FABP4

Participants’ baseline demographic characteristics, PD vintage, blood pressure (BP), body mass index (BMI), the presence of diabetes mellitus, and the medication data were collected by reviewing their medical records. Biochemical assays [serum creatinine, albumin, calcium, phosphate, alkaline phosphatase (ALP), fasting blood glucose, glycosylated hemoglobin (HbA1c), and lipids profile] were performed in accordance with the standard laboratory procedure, using an automated analyzer. The intact parathormone (iPTH) level was measured by the electrochemiluminescence immunoassay (Roche e801). Serum ultra-sensitive C-reactive protein (us-CRP) was detected by rate nephelometry (AU5800). Kt/V urea was calculated using the Daugirdas formula. The history of cardiovascular disease was recorded, including coronary artery diseases (myocardial infarction, angina pectoris, percutaneous coronary angioplasty, and coronary artery bypass surgery), congestive heart failure, stroke, and peripheral vascular diseases (angioplasty, vascular surgery, and amputation).

Blood samples were collected after fasting for 12 h. Serum FABP4 level was measured using a commercially available enzyme-linked immunosorbent assay (Phoenix Pharmaceutica, Burlingame, CA) according to the manufacturer’s instructions. Participants were classified into two groups according to the median value of serum FABP4: those with low FABP4 levels (<150.1 ng/mL) and those with high FABP4 levels (≥150.1 ng/mL).

Serum phosphate >1.45 mmol/L was defined as hyperphosphatemia, and serum corrected calcium >2.54 mmol/L was defined as hypercalcemia in the present study, in accordance with the target ranges of serum phosphate and corrected calcium for CKD stage 5D patients proposed by the 2017 KDIGO guideline.

### Body composition measurement

One hundred and ten patients finished the body composition measurement. Participants’ body composition was measured using the Body Composition Monitor (BCM, Fresenius Medical Care AG&CO., KGaA D-61346 Bad Homburg, Germany). We collected data including skeletal muscle mass (kg), body fat mass (kg), body fat percentage (%), lean body mass (kg), and overhydration value (OH, L).

### Assessment of AAC

Lateral lumbar radiography was used to evaluate patients’ AAC. The AAC score was calculated by two physicians using Kauppila scoring [12]. The AAC score ≥4 was considered to be a high AAC score, indicating moderate or heavy calcification [13].

### Statistical analysis

Statistical analyses were performed using SPSS version 22.0 (IBM, Armonk, NY). Normally distributed variables were expressed as mean ± standard deviation, and non-normally distributed variables were expressed as median with 25th and 75th percentiles. Categorical variables were presented as absolute (*n*) and percentages (%). To test the difference between two groups, independent Student’s *t*-test was used for normally distributed variables, the Mann–Whitney *U* test for non-normally distributed variables, and *χ*^2^ test for categorical variables. Correlation analysis was performed using the Spearman’s rank correlation method. Univariate and multivariate linear regression analyses were used to find the factors independently associated with the AAC score. Univariate and multivariate logistic regression analyses were used to find the independent influencers of an AAC score ≥4. Variables with *p*-value <0.1 in the univariate regression analysis and without collinearity relationships were selected into the multivariate regression analysis. A *p*-value <0.05 was considered statistically significant.

## Results

### Comparison of characteristics between the low FABP4 group and the high FABP4 group

The baseline characteristics are depicted in Table 1. One hundred and sixteen PD patients were enrolled in the study, including 56 men and 60 women. The mean age was 57.0 ± 13.7 years and the mean PD vintage was 50.0 (interquartile range 17.0–87.0) months. The median FABP4 level in these patients was 150.1 ng/mL.

**Table 1.** Comparison of clinical characteristics in accordance with serum FABP4 level.

Characteristics	All patients (*n* = 116)	FABP4 < 150.1ng/mL (*n* = 58)	FABP4 ≥ 150.1ng/mL (*n* = 58)	*p* value
Age (years)	57.0 ± 13.7	56.7 ± 15.5	57.2 ± 11.8	0.845
Male (*n*)	56 (48.3%)	31 (53.4%)	25 (43.1%)	0.265
Diabetic mellitus (*n*)	37 (31.9%)	26 (44.8%)	11 (19.0%)	0.003
History of cardiovascular disease (*n*)	47 (40.5%)	20 (34.5%)	27 (46.6%)	0.186
PD vintage (months)	50.0 (17.0, 87.0)	34.0 (7.0, 65.0)	63.5 (32.0, 93.0)	0.003
BMI (kg/m^2^)	23.4 ± 3.7	22.8 ± 4.0	24.1 ± 3.3	0.064
Systolic BP (mmHg)	133.6 ± 16.1	137.2 ± 13.2	129.9 ± 17.9	0.014
Diastolic BP (mmHg)	79.5 ± 12.3	80.3 ± 12.3	78.7 ± 12.2	0.465
Dialysate glucose load (g/d)	99.9 (88.6, 109.8)	97.2 (81.0, 109.8)	103.2 (97.2, 113.0)	0.088
Total Kt/V urea (per week)	1.83 (1.57, 2.16)	2.03 (1.72, 2.30)	1.72 (1.50, 1.87)	0.002
Residual kidney Kt/V urea (per week)	0.96 (0.00, 0.55)	0.31 (0.00, 0.76)	0.00 (0.00, 0.21)	0.002
Hemoglobin (g/L)	116.4 ± 16.2	116.7 ± 16.0	116.0 ± 16.5	0.824
Serum us-CRP (mg/L)	3.32 (0.89, 9.29)	1.73 (0.63, 7.01)	4.44 (2.08, 12.92)	0.003
Albumin (g/L)	37.4 ± 3.9	37.1 ± 3.6	37.8 ± 4.2	0.312
Creatinine (μmol/L)	931.5 ± 253.0	878.0 ± 261.5	985.0 ± 234.4	0.022
Corrected calcium (mmol/L)	2.53 ± 0.23	2.52 ± 0.21	2.54 ± 0.25	0.717
Phosphate (mmol/L)	1.63 ± 0.41	1.56 ± 0.39	1.70 ± 0.42	0.072
Ca × P (mg^2^/dL^2^)	51.0 ± 13.0	48.6 ± 11.5	53.4 ± 14.1	0.048
iPTH (pg/mL)	158.6 (85.1, 325.9)	142.4 (74.3, 232.1)	219.2 (99.1, 416.4)	0.007
ALP (U/L)	69.0 (57.0, 86.0)	63.5 (55.5, 74.5)	77.0 (59.8, 104.8)	0.003
Fasting blood glucose (mmol/L)	5.6 (4.8, 6.7)	5.7 (4.9, 9.0)	5.5 (4.8, 6.1)	0.209
HbA1c (%)	6.1 (5.5, 7.0)	6.4 (5.5, 7.4)	5.9 (5.2, 6.3)	0.185
TCHO (mmol/L)	4.78 ± 1.10	4.64 ± 0.98	4.91 ± 1.21	0.176
TG (mmol/L)	1.80 (1.34, 2.97)	1.57 (1.12, 2.64)	2.29 (1.48, 3.35)	0.003
HDL-C (mmol/L)	0.97 (0.83, 1.13)	0.98 (0.86, 1.16)	0.97 (0.79, 1.11)	0.229
LDL-C (mmol/L)	2.78 ± 0.77	2.74 ± 0.68	2.81 ± 0.85	0.609
Body composition (*n* = 110)				
Lean body mass (kg)	38.0 ± 9.9	39.2 ± 9.5	36.7 ± 10.2	0.187
Skeletal muscle mass (kg)	35.2 ± 9.1	36.2 ± 8.6	34.3 ± 9.5	0.277
Body fat mass (kg)	24.6 ± 11.0	22.1 ± 11.3	26.8 ± 10.2	0.024
Body fat percentage (%)	38.5 ± 14.3	34.8 ± 13.7	42.0 ± 14.0	0.007
Overhydration value (OH, L)	1.8 (0.8, 2.8)	2.1 (1.0, 2.9)	1.5 (0.7, 2.6)	0.067
Statin use, *n*	57 (49.1%)	31 (53.4%)	26 (44.8%)	0.353
Calcium carbonate use, *n*	71 (61.2%)	34 (53.5%)	37 (63.8%)	0.568
Non-calcium-containing phosphate binder use, *n*	41 (35.3%)	20 (34.5%)	21 (36.2%)	0.846
Calcitriol use, *n*	19 (16.4)	8 (13.8%)	11 (19.0%)	0.452
Serum FABP4 (ng/mL)	150.1 (131.6, 223.3)	131.6 (112.9, 140.2)	222.3 (169.6, 388.9)	＜0.001
AAC score	4.0 (1.0, 10.0)	3.0 (1.0, 6.0)	9.0 (2.0, 13.0)	<0.001
AAC score ≥4 (*n*)	61 (52.6%)	24 (41.4%)	37 (63.8%)	0.016

FABP4: fatty acid binding protein 4; PD: peritoneal dialysis; BMI: body mass index; BP: blood pressure; Kt/V urea: fractional urea clearance; us-CRP: ultra-sensitive C reactive protein; iPTH: intact parathyroid hormone; ALP: alkaline phosphatase; HbA1c: glycosylated hemoglobin; TCHO: total cholesterol; TG: triglyceride; HDL-C: high density lipoprotein cholesterol; LDL-C: low density lipoprotein cholesterol; AAC: abdominal aortic calcification.

The AAC score and the proportion of patients with an AAC score ≥4 of the high FABP4 group were higher than those of the low FABP4 group. The high FABP4 group had a longer PD vintage than the low FABP4 group. Serum us-CRP, creatinine, iPTH, ALP, calcium-phosphate product, and triglyceride (TG) of the high FABP4 group were higher than those of the low FABP4 group. Moreover, the high FABP4 group tended to have higher serum phosphate than the low FABP4 group though the difference was not significant.

The proportion of patients with diabetic mellitus, systolic BP, total Kt/V, and residual kidney Kt/V of the high FABP4 group were lower than those of the low FABP4 group. There were no significant differences in age, gender, BMI, dialysate glucose load, diastolic BP, hemoglobin, albumin, fasting blood glucose, HbA1c, corrected calcium, total cholesterol (TCHO), high density lipoprotein cholesterol (HDL-C), low density lipoprotein cholesterol (LDL-C), and medications between the two groups (Table 1).

Body composition analysis of the participants showed that the high FABP4 group had higher body fat mass and body fat percentage than the low FABP4 group. There were no significant differences in OH, lean body mass, and skeletal muscle mass between the two groups (Table 1).

### Comparison of characteristics between patients with an AAC score <4 and patients with an AAC score ≥4

Sixty-one patients (52.6%) were found to have an AAC score ≥4. As shown in Table 2, the high AAC score group had higher serum FABP4 [164.5 (138.4, 362.8) ng/mL versus 144.7 (123.8, 170.1) ng/mL, *p* = 0.002)] than that of the low AAC score group. The high AAC score group was older and had a longer PD vintage and more use of statins than the low AAC score group. The proportions of patients with diabetic mellitus, history of cardiovascular disease, serum phosphate >1.45 mmol/L, and corrected calcium >2.54 mmol/L of the high AAC score group were higher than those of the low AAC score group. Serum us-CRP, corrected calcium, body fat mass, and body fat percentage of the high AAC score group were also higher than those of the low AAC score group. Serum ALP of the high AAC score group tended to be higher than that of the low AAC score group, but the difference was not significant. Whereas, diastolic BP and serum albumin of the high AAC score group were lower than those of the low AAC score group (Table 2).

**Table 2.** Comparison of clinical characteristics among PD patients with an AAC score <4 or ≥4.

Characteristics	AAC score <4 (*n* = 55)	AAC score ≥4 (*n* = 61)	*p* value
Age (years)	51.7 ± 13.0	61.7 ± 12.7	<0.001
Male (*n*)	26 (47.3%)	30 (49.2%)	0.837
Diabetic mellitus (*n*)	12 (21.8%)	25 (41.0%)	0.027
History of cardiovascular disease	13 (23.6%)	34 (55.7%)	<0.001
PD vintage (months)	43.0 (12.0, 91.0)	51.0 (27.0, 83.5)	0.488
Dialysate glucose load (g/d)	97.2 (81.0, 109.8)	109.8 (97.2, 109.8)	0.088
Total Kt/V urea (per week)	1.83 (1.67, 2.16)	1.82 (1.54, 2.17)	0.616
Residual kidney Kt/V urea (per week)	0.19 (0.00, 0.69)	0.00 (0.00, 0.45)	0.236
Systolic BP (mmHg)	135.5 ± 15.1	131.8 ± 16.8	0.214
Diastolic BP (mmHg)	84.3 ± 10.0	75.1 ± 12.5	<0.001
us-CRP (mg/L)	1.56 (0.63, 4.87)	7.15 (2.30, 15.25)	<0.001
Albumin (g/L)	38.6 ± 3.4	36.3 ± 4.0	0.001
Fasting blood glucose (mmol/L)	5.3 (4.7, 5.9)	5.7 (5.1, 7.2)	0.097
HbA1c (%)	6.0 (5.1, 7.1)	6.1 (5.6, 6.9)	0.660
Corrected calcium (mmol/L)	2.49 ± 0.22	2.57 ± 0.23	0.045
Corrected calcium > 2.54 mmol/L (*n*)	19 (34.5%)	36 (59.0%)	0.008
Phosphate (mmol/L)	1.63 ± 0.46	1.62 ± 0.37	0.909
Phosphate >1.45 mmol/L (*n*)	30 (54.5%)	45 (73.8%)	0.031
Ca × P (mg^2^/dL^2^)	50.1 ± 13.4	51.9 ± 12.8	0.464
iPTH (pg/mL)	157.4 (70.1, 333.5)	159.8 (101.0, 316.8)	0.569
ALP (U/L)	67.0 (55.0, 79.0)	73.0 (59.5, 107.0)	0.054
TCHO (mmol/L)	4.72 ± 0.91	4.82 ± 1.26	0.610
TG (mmol/L)	1.62 (1.35, 2.62)	2.36 (1.33, 3.24)	0.148
HDL-C (mmol/L)	0.98 (0.86, 1.17)	0.95 (0.80, 1.11)	0.102
LDL-C (mmol/L)	2.80 ± 0.76	2.75 ± 0.78	0.704
Body composition (*n* = 110)			
Lean body mass (kg)	39.3 ± 10.0	36.9 ± 9.8	0.208
Skeletal muscle mass (kg)	37.1 ± 9.6	33.7 ± 8.4	0.052
Body fat mass (kg)	22.3 ± 10.8	26.4 ± 10.9	0.048
Body fat percentage (%)	35.4 ± 14.7	41.1 ± 13.5	0.035
Overhydration value (OH, L)	1.9 (0.9, 2.9)	1.8 (0.8, 2.8)	0.851
Statin use, *n*	16 (29.1%)	41 (67.2%)	<0.001
Calcium carbonate use, *n*	35 (63.6%)	36 (59.0%)	0.523
Non-calcium-containing phosphate binder use, *n*	20 (36.4%)	21 (34.4%)	0.827
Calcitriol use, *n*	8 (14.5%)	11 (18.0%)	0.612
AAC score	1.0 (0.0, 2.0)	9.0 (7.0, 13.0)	<0.001
Serum FABP4 (ng/mL)	144.7 (123.8, 170.1)	164.5 (138.4, 362.8)	0.002

PD: peritoneal dialysis; AAC: abdominal aortic calcification; Kt/V: fractional urea clearance; BP: blood pressure; us-CRP: ultra-sensitive C reactive protein; HbA1c: glycosylated hemoglobin; iPTH: intact parathyroid hormone; ALP: alkaline phosphatase; TCHO: total cholesterol; TG: triglyceride; HDL-C: high density lipoprotein cholesterol; LDL-C: low density lipoprotein cholesterol; FABP4: fatty acid binding protein 4.

### Correlation analysis of serum FABP4, AAC score, and clinical parameters

**Figure 1.**
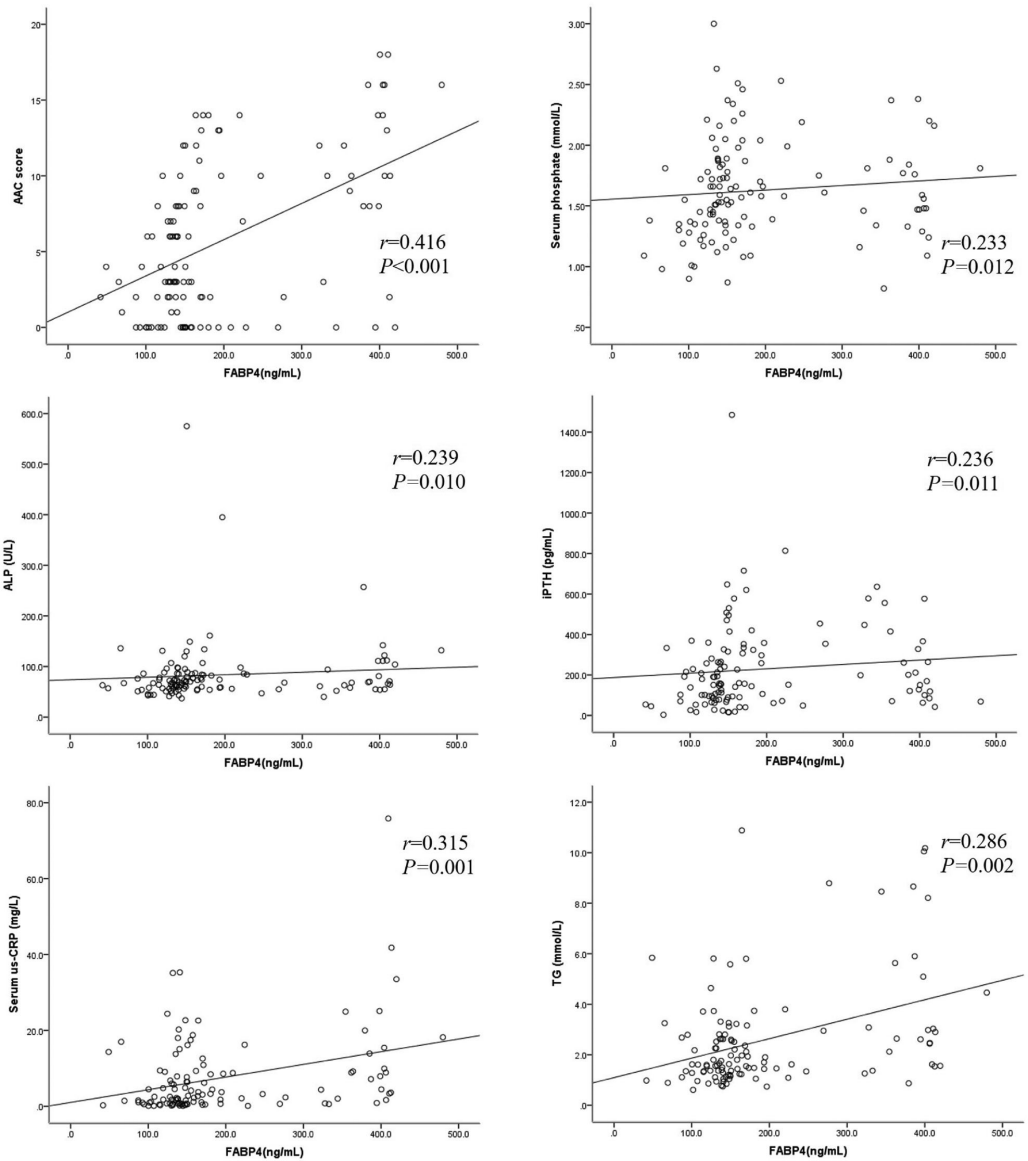
Correlations between serum FABP4, AAC score, and other clinical parameters.

Table 3 and Figure 1 show the correlations between serum FABP4, AAC score, and other clinical parameters in PD patients. The AAC score was positively associated with serum FABP4 (*r* = 0.416, *p* < 0.001; Figure 1), age, serum corrected calcium, us-CRP, TG, body fat mass, and body fat percentage. Meanwhile, the AAC score was negatively associated with diastolic BP, residual kidney Kt/V, albumin, HDL-C, skeletal muscle mass, and lean body mass.

**Table 3.** Correlations between the AAC score, serum FABP4 level, and other parameters.

Variables	AAC score		Serum FABP4 (ng/mL)	
	*r*	*p value*	*r*	*p value*
Age (years)	0.423	<0.001	0.033	0.728
PD vintage (months)	0.110	0.240	0.304	0.001
Systolic BP (mmHg)	−0.120	0.200	−0.205	0.027
Diastolic BP (mmHg)	−0.375	<0.001	−0.133	0.157
Total Kt/V urea (per week)	−0.174	0.107	−0.343	0.001
Residual kidney Kt/V urea (per week)	−0.293	0.006	−0.375	<0.001
BMI (kg/m^2^)	0.164	0.080	0.229	0.014
Fasting blood glucose (mmol/L)	0.151	0.107	−0.063	0.500
HbA1c (%)	0.042	0.750	−0.094	0.471
Hemoglobin (g/L)	0.018	0.845	0.018	0.850
Albumin (g/L)	−0.259	0.005	0.086	0.356
Creatinine (μmol/L)	−0.042	0.653	0.270	0.003
Corrected calcium (mmol/L)	0.189	0.042	0.035	0.708
Phosphate (mmol/L)	0.019	0.841	0.233	0.012
Ca × P (mg^2^/dL^2^)	0.079	0.398	0.227	0.014
TG (mmol/L)	0.237	0.010	0.286	0.002
HDL-C (mmol/L)	−0.206	0.027	−0.155	0.096
LDL-C (mmol/L)	−0.023	0.809	−0.002	0.985
TCHO (mmol/L)	0.059	0.528	0.156	0.095
ALP (U/L)	0.224	0.016	0.239	0.010
iPTH (pg/mL)	0.060	0.522	0.236	0.011
us-CRP (mg/L)	0.405	<0.001	0.315	0.001
Serum FABP4 (ng/mL)	0.416	<0.001	–	–
Skeletal muscle mass (kg)	−0.265	0.005	−0.171	0.075
Body fat mass (kg)	0.266	0.005	0.286	0.002
Body fat percentage (%)	0.293	0.002	0.312	0.001
Lean body mass (kg)	−0.223	0.019	−0.193	0.043
Overhydration value (OH, L)	−0.002	0.981	−0.250	0.011

AAC: abdominal aortic calcification; FABP4: fatty acid binding protein 4; PD: peritoneal dialysis; BP: blood pressure; Kt/V urea: fractional urea clearance; BMI: body mass index; HbA1c: glycosylated hemoglobin; TG: triglyceride; HDL-C: high density lipoprotein cholesterol; LDL-C: low density lipoprotein cholesterol; TCHO: total cholesterol; ALP: alkaline phosphatase; iPTH: intact parathyroid hormone; us-CRP: ultra-sensitive C reactive protein.

Serum FABP4 level was positively correlated with PD vintage, BMI, serum phosphate (Figure 1), us-CRP (Figure 1), TG (Figure 1), ALP (Figure 1), creatinine, calcium–phosphate product, body fat mass, and body fat percentage. Moreover, serum FABP4 was negatively correlated with systolic BP, total Kt/V, residual kidney Kt/V, OH, and lean body mass.

### Linear regression analysis for the AAC score

Univariate linear regression analyses demonstrated that serum FABP4, age, the history of cardiovascular disease, serum corrected calcium, corrected calcium >2.54 mmol/L, us-CRP, TG, body fat mass, body fat percentage, and lean body mass were positively associated with the AAC score (*p* < 0.1). However, systolic BP, diastolic BP, residual kidney Kt/V, albumin, HDL-C, and skeletal muscle mass were negatively associated with the AAC score in PD patients (*p* < 0.1).

Variables with *p*-value <0.1 in the univariate analysis and without collinearity issues were included in the multivariate linear regression analysis (corrected calcium >2.54 mmol/L, body fat percentage, and lean body mass were not included). Serum FABP4, age, and albumin remained significantly associated with the AAC score in PD patients after adjustment for the history of cardiovascular disease, systolic BP, diastolic BP, residual kidney Kt/V, serum corrected calcium, us-CRP, TG, HDL-C, skeletal muscle mass, and body fat mass (Table 4).

**Table 4.** Univariate and multivariate linear regression analysis for the AAC score.

Parameters	Univariate linear regression		Multivariate linear regression	
	*β*	*p value*	*β*	*p value*
Age (years)	0.135	<0.001	0.100	0.007
History of cardiovascular disease (%)	2.990	0.002	Not selected	–
Systolic BP (mmHg)	−0.059	0.047	Not selected	–
Diastolic BP (mmHg)	−0.158	<0.001	Not selected	–
Residual kidney Kt/V urea (peer week)	−3.501	0.011	Not selected	–
Albumin (g/L)	−0.282	0.021	−0.291	0.020
Corrected calcium (mmol/L)	4.220	0.042	Not selected	–
TG (mmol/L)	0.714	0.001	Not selected	–
HDL-C (mmol/L)	−4.235	0.016	Not selected	–
us-CRP (mg/L)	0.134	0.002	Not selected	–
Serum FABP4 (ng/mL)	0.024	<0.001	0.026	<0.001
Skeletal muscle mass (kg)	−0.159	0.003	Not selected	–
Body fat mass (kg)	0.140	0.001	Not selected	–

AAC: abdominal aortic calcification; BP: blood pressure; Kt/V urea: fractional urea clearance; TG: triglyceride; HDL-C: high density lipoprotein cholesterol; us-CRP: ultra-sensitive C reactive protein; FABP4: fatty acid binding protein 4.

### Independent influencers of an AAC score ≥4 by logistic analysis

Univariate logistic regression analyses showed that serum FABP4, age, diabetic mellitus, the history of cardiovascular disease, diastolic BP, albumin, ALP, us-CRP, serum phosphate, serum phosphate >1.45 mmol/L, serum corrected calcium, corrected calcium >2.54 mmol/L, skeletal muscle mass, body fat mass, and body fat percentage were associated with an AAC score ≥4 (*p* < 0.1).

Variables with *p*-value <0.1 in the univariate analysis and without collinearity issues were included in the multivariate logistic regression analysis to confirm the independent influencers of an AAC score ≥4 (serum phosphate, serum corrected calcium, and body fat percentage were not included). Finally, serum FABP4, age, diabetic mellitus, serum albumin, serum corrected calcium >2.54 mmol/L, and serum phosphate >1.45 mmol/L were independently associated with an AAC score ≥4 after adjustment for the history of cardiovascular disease, diastolic BP, ALP, us-CRP, skeletal muscle mass, and body fat mass (Table 5).

**Table 5.** Independent factors associated with an AAC score ≥4 by logistic regression analysis.

Parameters	Univariate		Multivariate	
	Exp (*B*) (95% CI)	*p value*	Exp (*B*) (95% CI)	*p value*
Age (years)	1.062 (1.029–1.096)	<0.001	1.134 (1.065–1.206)	<0.001
Diabetic mellitus	2.488 (1.098–5.641)	0.029	34.578 (5.885–203.161)	<0.001
History of cardiovascular disease (%)	4.068 (1.826–9.067)	0.001	Not selected	–
Diastolic BP (mmHg)	0.928 (0.892–0.965)	<0.001	Not selected	–
Albumin (g/L)	0.841 (0.754–0.940)	0.002	0.788 (0.636–0.976)	0.029
Corrected calcium >2.54 mmol/L	2.728 (1.283–5.802)	0.009	3.982 (1.150–13.782)	0.029
Phosphate >1.45 mmol/L	2.344 (1.075–5.109)	0.032	10.766 (2.699–42.939)	0.001
ALP (U/L)	1.016 (1.001–1.031)	0.037	Not selected	–
TG (mmol/L)	1.237 (1.005–1.523)	0.037	Not selected	–
HDL-C (mmol/L)	0.231 (0.051–1.052)	0.058	Not selected	–
us-CRP (mg/L)	1.092 (1.030–1.158)	0.003	Not selected	–
Serum FABP4 (ng/mL)	1.006 (1.002–1.010)	0.003	1.015 (1.007–1.023)	<0.001
Skeletal muscle mass (kg)	0.958 (0.918–1.001)	0.055	Not selected	–
Body fat mass (kg)	1.036 (1.000–1.074)	0.051	Not selected	–

AAC: abdominal aortic calcification; BP: blood pressure; ALP: alkaline phosphatase; TG: triglyceride; HDL-C: high density lipoprotein cholesterol; us-CRP: ultra-sensitive C reactive protein; FABP4: fatty acid binding protein 4.

## Discussion

In this study, serum FABP4 levels were positively associated with the AAC score by lateral abdominal X-ray plain films. Serum FABP4 level was the independent influencer of an AAC score ≥4, indicating moderate or heavy VC in PD patients. The results raised FABP4 as a novel biomarker and potential treating target of VC in PD patients.

Our study is the first to identify the positive association between serum FABP4 and VC in PD patients. The AAC score was positively associated with TG, us-CRP, body fat mass, and body fat percentage. FABP4 was secreted mainly by adipose tissues and macrophages. The abundant source among PD patients contributed to the positive association between FABP4 and VC. Besides, FABP4 may affect PD patients’ atherosclerotic intimal calcification. Serum FABP4 was significantly associated with TG, BMI, and body fat mass in the study. Previous studies have demonstrated that circulating FABP4 positively correlated with obesity [14], hypertension [15], dyslipidemia [16], insulin resistance [17], waist circumference [18], and atherosclerosis [19], all of which are traditional risk factors of cardiovascular disease (CVD). Accordingly, a human study suggested that reduced expression of FABP4 in adipose tissue had beneficial effects on CVD [20].

On the other hand, our results showed that circulating FABP4 was negatively associated with residual kidney Kt/V, and the high FABP4 group had significantly lower residual kidney Kt/V than the low FABP4 group (*p* = 0.002). FABP4 appears to be accumulated in circulation due to the diminished renal excretion in patients with ESRD.

Glucose absorption from dialysate, diet, and medications are all contributors of the abnormal lipid metabolism in PD patients. In the present study, the high AAC score group had higher TG and body fat mass, but lower serum albumin than the low AAC score group. Hypoalbuminemia is an important element of malnutrition in dialysis patients, and our results showed that it may coexist with abnormal fat metabolism and induce VC in PD patients together. Recently Sung et al. [21] reported that FABP4 levels were positively associated with central arterial stiffness, which is closely related with VC in PD patients. PD patients may have more complicate calcium balance status and more excessive fat accumulation than hemodialysis patients. Circulating FABP4 was also found to be positively associated with several metabolic parameters in hemodialysis patients [22]. Furuhashi et al. [23] suggested serum FABP4 as a novel predictor of the cardiovascular mortality in patients with ESRD. Furthermore, FABP4 could be locally produced by the perivascular fat and macrophages in the vascular plaques and contributed to the development of coronary atherosclerosis [24]. It was reported that FABP4 could induce the proliferation and migration of human coronary artery smooth muscle cells through a mitogen activated protein kinase (MAPK)-dependent pathway [25].

Medial arterial calcification is more specific than intimal calcification in patients with CKD-MBD, in which process FABP4 might also be involved. The traditional risk factors of medial arterial calcification include older age, hypercalcemia, hyperphosphatemia, prolonged dialysis vintage, and inflammation. First, FABP4 could induce the activation of oxidative stress in endothelial cells [26]. The activation of insulin-signaling pathway was inhibited by exogenous FABP4 in basic studies, resulting in decreased endothelial nitric oxide synthase (eNOS) activation and nitric oxide (NO) production. Second, FABP4 may increase inflammatory reactions. FABP4 downregulated the expression of sirtuin 3 [27], uncoupling protein 2 [28], and peroxisome proliferator receptor-γ coactivator 1α in the macrophages. These proteins led to increased reactive oxygen species (ROS) generation [29]. Accordingly, we found that serum FABP4 positively correlated with serum us-CRP in PD patients. Obviously, more study is needed to test the possible role of FABP4 in the inflammatory response among PD patients.

Another interesting finding was that serum FABP4 positively correlated with serum phosphate, and serum phosphate >1.45 mmol/L was independently associated with severe AAC in the study. Hyperphosphatemia is the main cause of VC in patients with ESRD. Literature on the relationship between FABP4 and phosphorus metabolism is rare. Peri-Okonny et al. [30] found that high inorganic phosphate diet downregulated *FABP4* expression in the muscle of mice, and impaired the uptake and transport of fatty acid in the skeletal muscle. Hyperphosphatemia induces the osteo-/chondrogenic transdifferentiation of vascular smooth muscle cells, and predominantly increases the medial calcification in CKD patients. The involved nuclear factor-kappaB and Wnt-β-catenin pathways could be regulated by FABP4 in animal studies [31]. Although no research has directly investigated the impact of FABP4 on phosphate metabolism in CKD patients, there were reports of other adipokines like leptin and adiponectin. Bone damage is an important cause of hyperphosphatemia in CKD-MBD. Wang et al. [9] reported that leptin was positively associated with bone mineral density (BMD) in hemodialysis patients. *In vitro*, leptin could stimulate the proliferation and differentiation of bone marrow mesenchymal stem cells to osteoblasts [32], inhibit osteoclastogenesis [33], and activate fibroblast grow factor-23 [34]. Serum adiponectin was found to be negatively associated with BMD in male hemodialysis patients [10]. Adiponectin may induce the proliferation and differentiation of human osteoblasts, increase osteoclast formation, and inhibit osteoprotegerin production in osteoblasts [35]. In this regard, we found that serum FABP4 positively correlated with iPTH and ALP, thus we speculate that elevated FABP4 may exacerbate the imbalance between bone formation and resorption, accentuate hyperphosphatemia, and consequently promote VC. Prospective studies are needed to confirm the relationship between FABP4 and bone metabolism.

Some factors are well-recognized predictors of VC in PD patients, such as residual kidney function, volume overload, and iPTH. In the current study, we found that residual kidney Kt/V was negatively associated with the AAC score in the univariate linear regression analysis, but it was not the independent influencer of the AAC score in the multivariate linear regression analysis. There was no significant association between OH and AAC in the study. We found no significant correlation between iPTH and VC, possibly due to the relatively small sample size, the well-controlled serum iPTH, and the single iPTH data used in the study.

The limitations of the present study include the relatively small sample size, the cross-sectional nature of both FABP4 and AAC measurements, and the lack of bone histomorphometric data. Residual confounding factors still remained possible even after adjustment for classical risk factors of VC.

In conclusion, our study is the first to show the positive association between serum FABP4 and the AAC score in PD patients. Higher serum FABP4 level was independently associated with an AAC score ≥4 in PD patients. These findings indicate that FABP4 could be used as a novel biomarker of VC for further investigation.

## Data Availability

The data underlying this article will be shared upon reasonable request to the corresponding author.
